# Reconfigurable Wireless Sensor Node Remote Laboratory Platform with Cloud Connectivity

**DOI:** 10.3390/s21196405

**Published:** 2021-09-25

**Authors:** Tinashe Chamunorwa, Horia Alexandru Modran, Doru Ursuțiu, Cornel Samoilă, Horia Hedeșiu

**Affiliations:** 1 Faculty of Electrical Engineering and Computer Science, Transilvania University of Brasov, 500036 Brasov, Romania; csam@unitbv.ro; 2 Romanian Academy of Scientists, 050044 Bucharest, Romania; 3 Romanian Academy of Technical Sciences, 010413 Bucharest, Romania; 4 Electrical Machines and Drives Department, Technical University of Cluj Napoca, 400027 Cluj-Napoca, Romania; horia.hedesiu@emd.utcluj.ro

**Keywords:** cloud, reconfigurable, tags, pmod sensors, myRIO, SystemLink Cloud

## Abstract

Thanks to the recent rapid technological advancement in IoT usage, there is a need for students to learn IoT-based concepts using a dedicated experimental platform. Furthermore, being forced into remote learning due to the ongoing COVID-19 pandemic, there is an urgent need for innovative learning methods. From our perspective, a learning platform should be reconfigurable to accommodate multiple applications and remotely accessible at any time, from anywhere, and on any connected device. Considering that many of the university courses are now held online, the reliability and scalability of the system become critical. This paper presents the design and development of a wireless configurable myRIO-based sensor node that connects to SystemLink Cloud. The sensors that were used are for ambient light, temperature, and proximity. A graphical programming environment (G-LabVIEW) and related APIs were used for rapid concept-to-development process. Distinct applications have been developed for the instructor and students, respectively. The students can select which sensor and application to run on the system and observe the measurements on the local student’s application or the cloud platform at a specific moment. They can also read the data on the cloud platform and use them in their LabVIEW application. In the context of remote education, we strongly believe that this platform is and will be suitable for the COVID and Post-COVID eras as well because it creates a much better remote laboratory experience for students. In conclusion, the system that was developed is innovative because it is software reconfigurable from the device, from the instructor’s application and cloud via a web browser, it is intuitive, and it has a user-friendly interface. It meets most of the necessary requirements in the current era, being also highly available and scalable in the cloud.

## 1. Introduction

Learning through experimentation is a very essential and helpful component of education today. Traditional laboratories from university or research centers are already used for this purpose, but they have the disadvantage of having a limited capacity to accommodate all students [1]. Because developing new university laboratory facilities require a large financial investment, online laboratories proved to be a viable alternative solution for the learning process in the current era.

Recent technological advances, which have influenced the development of software and hardware, as well as networking and software, provide an excellent environment for the development of online remote laboratories, which can successfully be applied in education [2,3]. Furthermore, due to the ongoing coronavirus (COVID-19) pandemic, when many of the university courses were held online, the reliability and scalability of the learning platform became critical.

Starting in the spring of 2020, the rapid spread of COVID-19 resulted in the emergence of the online mode of learning strategy. While theory classes were mostly conducted online, universities were facing difficulties in conducting laboratory or practical classes, which are an integral component in engineering, causing stress in the higher education institutions [4]. The research conducted by Radhamani et al. [4] analyzes the switching intention of students from conventional education to remote education. It also explores the complements of physical experiments brought in with remote laboratory set-ups in the educational process. By comparing the usage of the learning platform in the academic year before COVID and those of the year 2020/2021, they concluded that usage of online/remote laboratories increased during the pandemic. The results suggest virtual laboratories may have an important role in inquiry-based education, which can play a crucial role in hands-on practice in the context of post-COVID-19 education.

The ongoing COVID-19 pandemic required engineering instructors to quickly rethink and redesign their courses to motivate and support students in a different remote learning environment. This adjustment was very challenging for courses with a laboratory component, where electronic hardware equipment had to be exchanged [5].

All the parties involved in this migration to remote learning must realize that these crises also create disruptions and inconvenience to both university staff and students [6].

One of the first generic remote engineering laboratories was developed in 1999 [7]. The proposed laboratory was accessible over a heterogeneous computer network, and it provided a learning environment where students could interact with instructors when performing experiments. It featured an incorporated stepper motor data acquisition system, which could be controlled by the students from their personal computer, over the Internet, for simple experiments.

By using cloud computing, the resources available on a physical machine could be shareable and interoperable. Furthermore, if the teaching materials are designed in such a way that the users are not concerned with technical issues, then the end-users (the students) will have a much better experience [8].

The purpose of the investigation is to develop an innovative remote learning laboratory based on cloud service that will enable instructors and students to collaborate for creating reconfigurable experiments using sensors.

The current paper is structured in five main sections, the first section being the introduction. The second section briefly presents the related work in terms of remote or cloud laboratories. Section 3 describes step by step the design and development for building the online learning platform with cloud connectivity. Section 4 shows the results of the experiment carried out, also providing some discussion and further steps, while in Section 5 are presented the conclusions. The paper concludes with the references used.

## 2. Related Work

Previous research in the field of remote laboratories proved that these platforms could improve students’ understanding of the lessons, sometimes being even more efficient than real laboratories due to wide accessibility and flexibility, as they can be accessed anytime, anywhere, and from any connected device. Furthermore, they are less expensive and can be used to teach a high number of students [9].

In the past few years, several scientific articles approached the topic of remote or virtual labs and, in this section, a short analysis and comparison on that topic in the context of the COVID-19 pandemic are provided. However, there are not many previous laboratories incorporating cloud connectivity.

The study conducted by Ramya et al. [1] illustrates the concept of the IoT based remote laboratory, which is used for sensor experiments. The authors are using the sensor module for controlling and monitoring the parameters of industrial equipment. They claim that, by using their platform, students can develop knowledge on sensors and then apply them to real industrial automation equipment [1].

The paper written by Zapata Rivera [2] presents an approach for developing a cloud-based smart adaptive remote laboratory. It can improve the students’ experience by adapting the experiments based on the student profile [2]. Even if all students access the same learning platform, the system might show a different user interface in accordance with the level of knowledge of the respective student. The remote laboratory features a webcam and a Raspberry Pi microcontroller. The software of the platform was developed using Python and JavaScript programming languages, and it has HTML 5 web user interface.

The approach proposed by Vitliemov et al. [8] for developing a remote lab contains some improvements over most remote laboratories, considering the architecture and scalability of the platform. The platform is based on a server storing learning resources that are static. The remote laboratories can be exchanged by several universities, where each organization maintains its own labs. Furthermore, the authors state that they can be operated by organizations with different goals and with different students’ experiences. This platform also has some downsides, and further work is necessary to ensure the unity of recent curricular materials [8].

In another paper that was analyzed [9], a virtual laboratory is used for digital systems courses that are taken online. The online experiment was carried out using the Breadboard simulator and the TeamViewer application. Their research was carried out with 20 engineering students as subjects. The results showed that the online laboratory platform had a positive perception from the students in terms of the educational aspect, the learning model, and means of operation, increasing the students’ motivation as well [9].

In the context of the COVID-19 pandemic, important changes have been imposed, which are mainly related to the structure, the mode of operation, and the operational phase that involve instructors and students [10]. The authors of this study believe that the most difficult part was the implementation of experimental activities through remote labs. They propose a possible implementation of a remote laboratory through the adoption of a software platform to connect students to the physical laboratory. The authors use an interconnection of LabVIEW, MATLAB, and Arduino to allow the students to carry out remote experiments from anywhere. Through this platform, the students can remotely perform tests and measurements, therefore validating the theory through experiments. They presented an experiment of analyzing the vibrations of a DC motor, using LabVIEW for the characterization, Arduino for data acquisition, and MATLAB [10].

The development of an efficient and innovative remote education system is considered one of the crucial requirements for the post-COVID-19 era [11]. Elmesalawy et al. [11] present the requirements and architectural design of a laboratory learning platform that can be used in different educational fields for performing experimental activities. An efficient and flexible system design has been proposed based on the needed requirements to support different types of remote-controlled laboratory experiments [11].

An important challenge of the COVID pandemic era was to deliver programming labs over the Internet without important methodological changes [12]. Most of the existing approaches to remote programming labs are based on asynchronous learning, where students work individually and can interact with the instructor. The authors defined an infrastructure that enables the delivery of synchronous programming labs over the Internet. After it was tested for both programming labs and exams, students expressed a high degree of satisfaction. The authors observed that the use of the system did not produce significant differences in student’s grades compared to previous years [12].

Although the current situation is very delicate, after it is over, universities will emerge with an important opportunity to became aware of the degree that they will be able to implement ERT to maintain continuity of educational processes [6].

Z. Zacharia [13] concluded that the manipulation of equipment, either physical or virtual, and no-touch sensory feedback is the important aspect of learning through science experimentation. Furthermore, he believes that researchers must identify when the sensory feedback must be present during students’ learning through experimentation to be able to provide the corresponding physical experiences that would enable students to gain the necessary knowledge for understanding certain concepts and definitions [13].

D. Vlachopoulos et al. [14] analyze the perceptions of online university staff about e-learning to define the concept and set the boundaries of this area. Their study provides a valuable definition, which enables other researchers to advance in the identification and analysis of how e-learning is carried out in different models and environments [14].

Due to recent advances on the Internet of Things (IoT), there are many facilities that can expand the experimental area in the field of remote and cloud laboratories in the current era.

## 3. Materials and Methods

The system developed can be used by both instructors and students for electronics education using sensors, but it can be extended to include more myRIO devices and various experiments, a phase which is still in development. It is innovative because it is software reconfigurable from the device, from the instructor’s application, and from the cloud via a web browser, and it is intuitive and has a user-friendly interface. The main advantages for which the use the cloud was adopted are that it is scalable, secure, and flexible, and it does not require maintenance. Furthermore, it offers the benefits of the Software as a Service (SaaS) delivery model, which incorporates the pay as you go option.

### 3.1. Hardware Implementation

**Figure 1:**
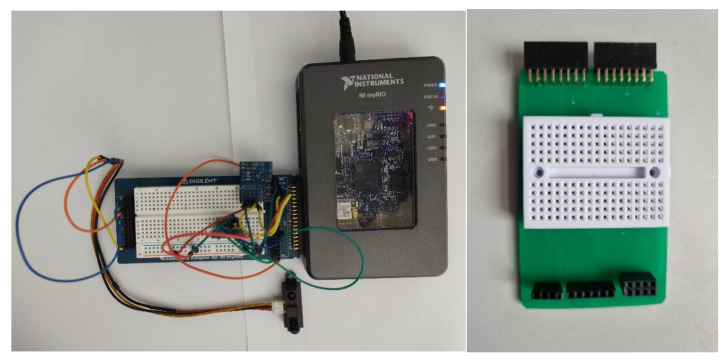
Hardware experimental setup.

Three integrated sensors were connected to the myRIO Student Embedded Device (Figure 1) [15]. The sensors are a SHARP GP2Y0A21YK0F IR range finder (IR sensor) [16], ambient light sensor (PmodALS) [17], and temperature sensor (PmodTMP3) [18]. The connections of these sensors to the myRIO device are detailed in [19]. Figure 1 shows the prototype breadboard (left) and the PCB developed using the Voltera PCB Printer (right), which is used to connect the sensors directly to myRIO.

### 3.2. myRIO-PC Wi-Fi Connection

The myRIO device was connected to the laptop over Wi-Fi. The procedure to setup myRIO as an access point is as follows:

- Launch LabVIEW and click the Set Up and Explore link.
- Select Configure Wi-Fi.
- Choose creating a wire network tab.
- Complete the task as shown on the displayed page.

### 3.3. LabVIEW Programming

**Figure 2:**
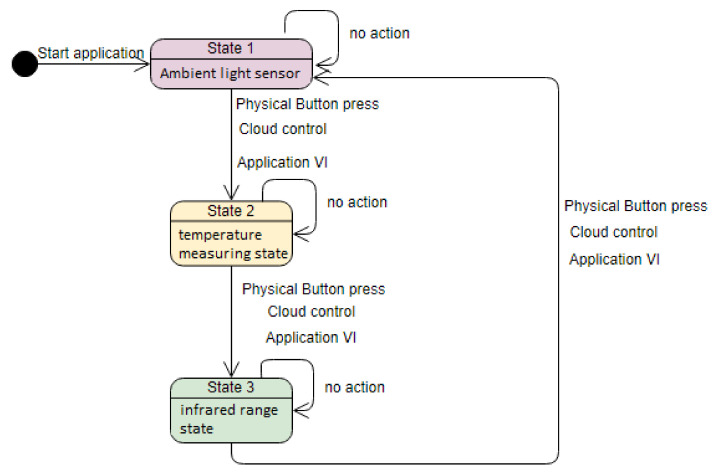
Finite state machine architecture.

A LabVIEW project was created and the myRIO device was added to the project explorer. The LabVIEW code is implemented using a finite state machine. Each state corresponds to a sensor connected to myRIO: the first state is for ambient light, the second is for temperature, and the last is for infrared range measurement. The architecture of the finite state machine is illustrated in Figure 2. There are three actions that enable the change from one state to another: a physical button press on myRIO device, a cloud control, and a button on the Front Panel of a Virtual Instrument.

**Figure 3:**
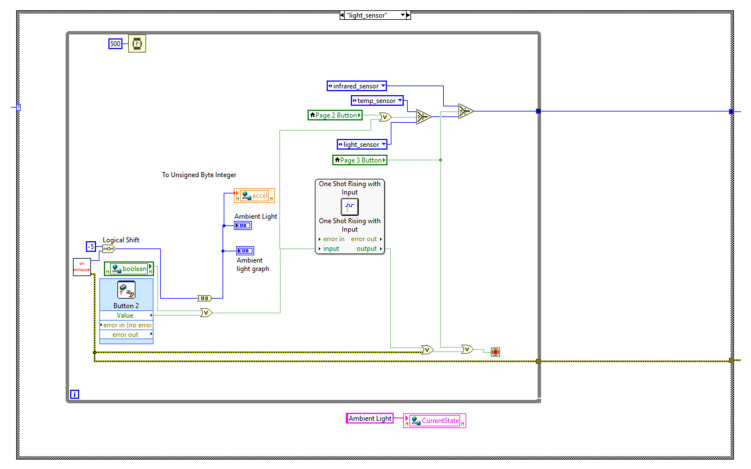
Ambient light measuring state.

**Figure 4:**
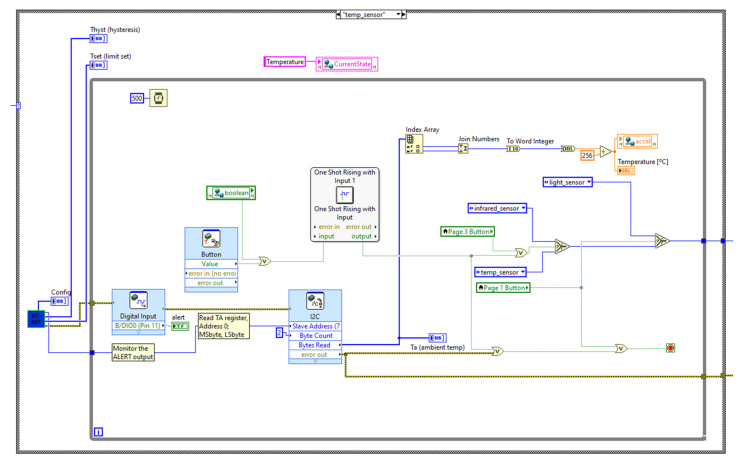
Temperature measuring state.

**Figure 5:**
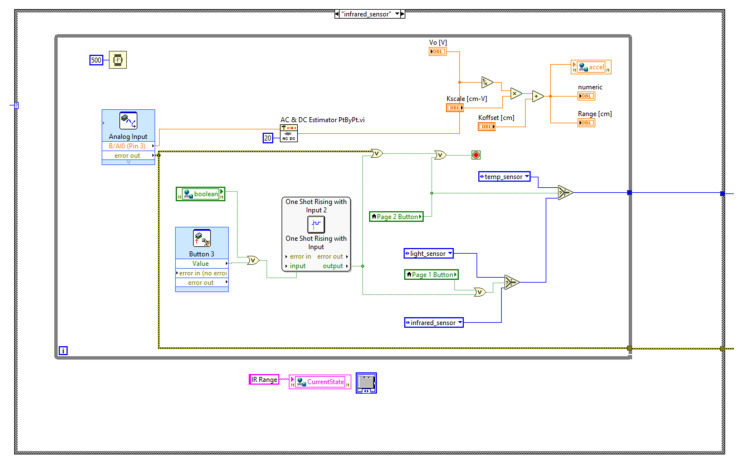
Infrared range measuring state.

The state machine code is placed under the myRIO device in the project hierarchy to enable code deployment directly into the device. Figure 3, Figure 4 and Figure 5 illustrate the block diagrams that corresponds to each state.

**Figure 6:**
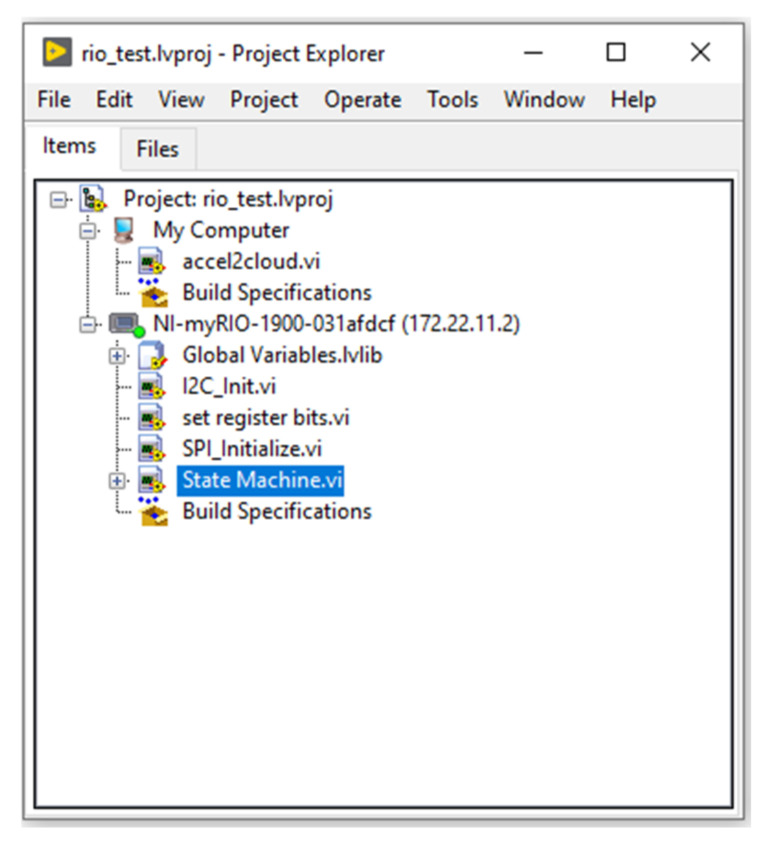
State machine VI project.

The VI that enables connection between myRIO and SystemLink Cloud is placed above the myRIO device in the project explorer hierarchy, and the acquired data are passed between the VIs through global variables. Figure 6 shows the hierarchy of the whole LabVIEW project.

A global variable is used to pass data from the state machine VI to the Virtual Instrument which sends the data to the cloud. The transmitting VI is placed above the myRIO device in the project explorer hierarchy.

To change between states, Button0 from the myRIO device and a Boolean switch on the SystemLink Cloud interface were configured. This enables the code to choose a sensor that transmits and stores its values in the global variable. The selection can be done either from the device or from the cloud platform.

Figure 3 illustrates the ambient light measuring state. The SPI Initialize subVI contains the ambient light sensing code [20]. In the light sensor state the ALS sensor gives output corresponding to the changes in the ambient light. The code will proceed to the next state on a physical button or web switch, otherwise it remains in the same state.

Figure 4 shows the temperature processing state. The I2C Init subVI contains part of the temperature measuring block diagram. The code will run in the temp_sensor state and only proceeds to the next when a button or switch is pressed. The VI measures the ambient temperature and stores the values in the global variable.

Figure 5 shows the Infrared measuring state. The AC and DC subVI contains part of the block diagram to measure the IR range. Any object can be used to vary the distance and observe the changes on the front panel.

### 3.4. myRIO Sytemlink Cloud Connection

The code that enables the connection is placed above the myRIO device in the LabVIEW project explorer hierarchy. This section covers the basics of using the LabVIEW to access data remotely from anywhere. Using LabVIEW, anyone can develop a program and publish it to the Internet to be hosted on the NI SystemLink Cloud.

The data stored in the global variable from the state machine VIs are then written to the cloud through the Tag that was created. The switch state from the cloud interface is also transmitted using a Boolean global variable to the state machine VI, to control the state machine actions.

The steps for creating the LabVIEW cloud connection VI are described in Table 1.

**Table 1:** Creating cloud connection.

Step	Action
1	In the Block Diagram, go to Data Communications -> SystemLink -> Configuration -> Open Configuration/Close Configuration.
2	Add an Open Configuration VI and Open Tag VI to the block diagram environment.
3	Configure the Open configuration VI to API Key as shown in Figure 5 (the server URL and API Key needs to be provided from SystemLink Cloud).
4	Create String Controls or Constants for server URL and API key.
5	The Open/Write/Read and Close Tag VI are found under: Data Communications -> SystemLink -> Tags.
6	Add an Open Tag VI and configure it as HTTP, then add constants for the data type and path.
7	The connection and the configuration for the first part of the VI is illustrated in Figure 5.
8	Go to Data Communications -> SystemLink -> Tags.
9	Add Write Tag.vi to the Block Diagram and configure it as Double data type to match the data type of the global variable.
10	Connect the rest of the VI inside a While loop as shown in Figure 5.
11	Provide the names of the path, which must match the name tags used (as shown in Figure 7).
12	For each tag, select the proper data type corresponding to data received from the cloud.
13	The top part of the VI, containing the Read Tag.vi, fetches the switch status from the cloud to LabVIEW through the Boolean global variable (Figure 8).
14	The names of the used Tag paths are light for switch and temp for sensor data.

**Figure 7:**
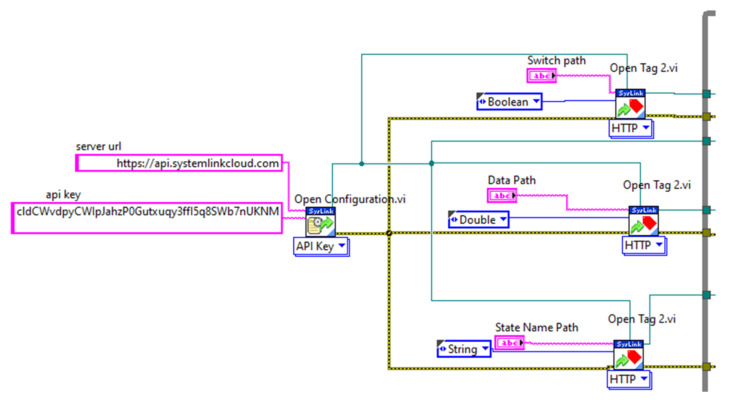
Open configuration and open tag VI connection.

**Figure 8:**
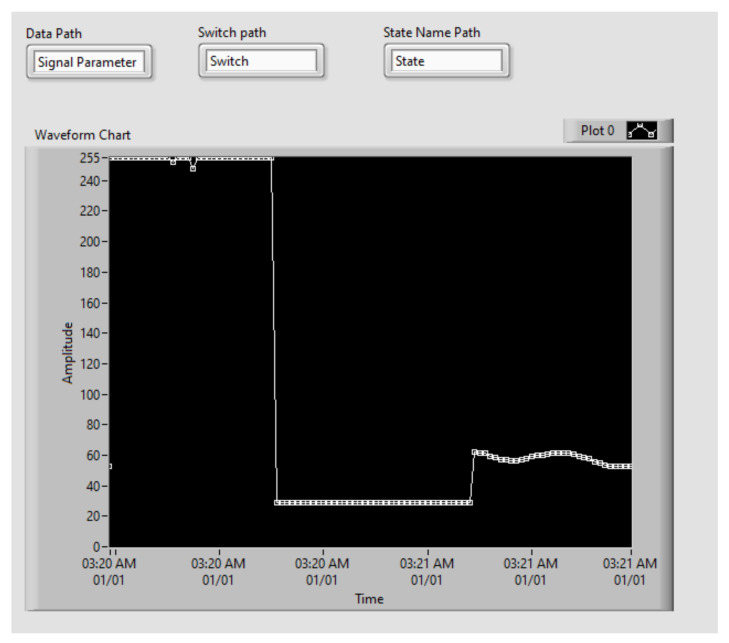
User interface with sample measurement.

**Figure 9:**
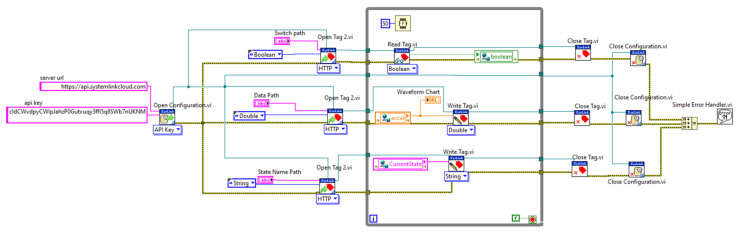
Whole VI of myRIO–cloud connection VI.

Figure 7 illustrates the opening configuration for the SystemLink Cloud, while the whole VI is pictured in Figure 9. A sample two-minute measurement for ambient light, followed by temperature and range is shown in Figure 8.

After the whole Block Diagram is completed and the program works fine, the next step would be to create the SystemLink Cloud interface [21].

**Figure 10:**
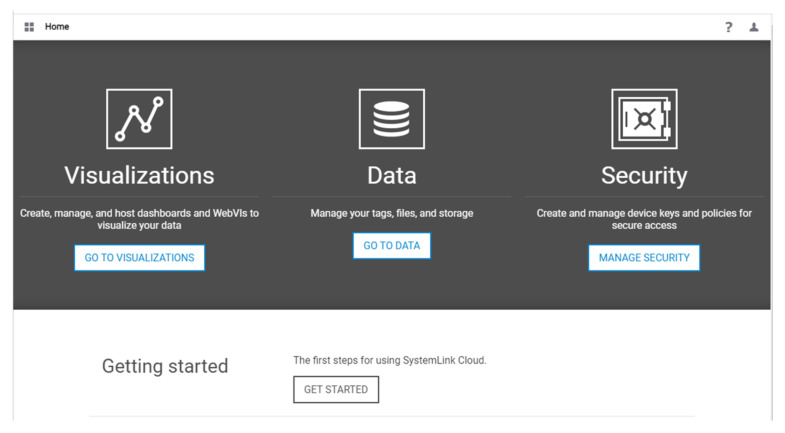
SystemLink Cloud dashboard.

For doing this, it is necessary to open the SystemLink Cloud interface through a web browser and log in using the NI account (Figure 10). The steps required for this process are detailed in Table 2.

**Table 2:** SystemLink Cloud configuration.

Step	Action
1	Click on Manage Security.
2	Select API keys and click on NEW to create a new key and copy the generated key. It is highly recommended to save it, as it will be provided only once.
3	Select on Policies and configure Policy settings. Click on Privileges and assign appropriate privileges as desired (Figure 11).
4	Assign the created policy to the API key.
5	Go to the Dashboard, click on Data and then Tags (Figure 12).
6	Create new Tag and assign appropriate data type.
7	Go to Visualizations and click on Dashboards to create a new dashboard. Choose a name and select the free-form type.
8	Add widget components (Table 3) to the dashboard according to the functionality of the LabVIEW program.
9	Drag an appropriate tag to a corresponding display or input widget.
10	Run the LabVIEW and the cloud Dashboard to see results.

**Figure 13:**
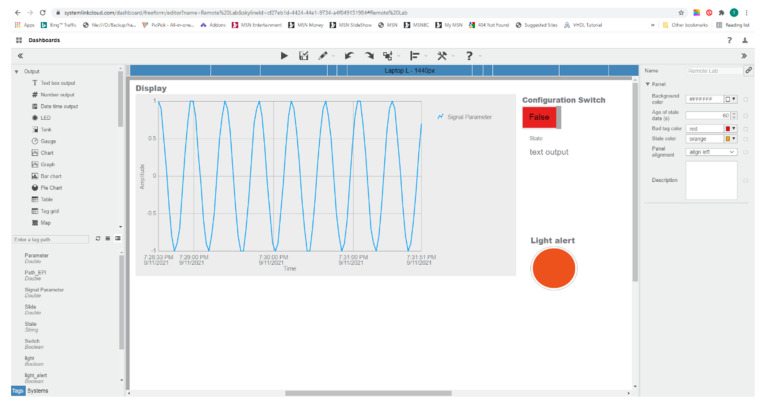
SystemLink dashboard configuration.

Figure 13 shows the dashboard web configuration interface. The interface is intuitive as it involves graphical and pictorial designing by the drag and drop technique.

Table 3 lists some of the configurable graphics available. These widgets can be customized to suit different applications and various data types.

## 4. Results and Discussion

A couple of learning experiments were conducted to test and confirm the functionality of the learning platform. In this process, the following features have been tested: the functionality of the sensor node, the reconfigurability of the system, and the cloud connectivity. Furthermore, an instructor and a student application were developed to interact with the remote laboratory.

**Figure 14:**
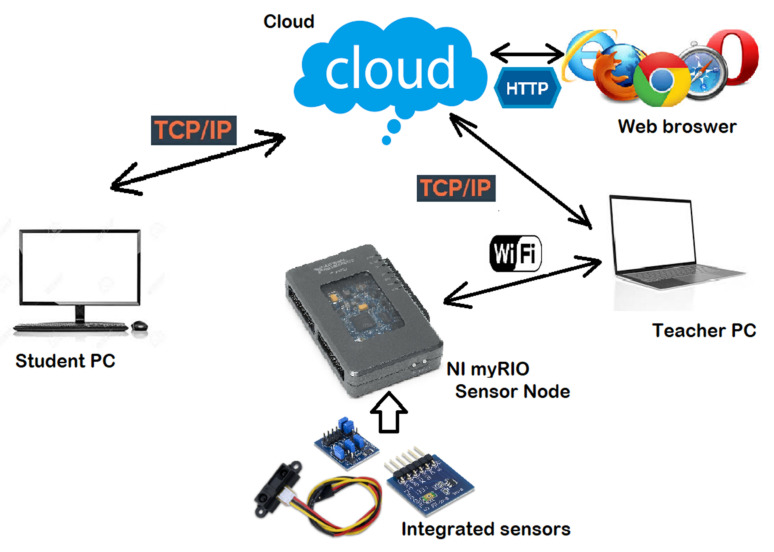
Overall architecture of the system.

The overall resulting architecture of our learning platform is illustrated in Figure 14.

The wireless myRIO sensor node can connect to the instructor’s PC and sends the data acquired to the cloud through LabVIEW. It can be reconfigured directly from the device, from LabVIEW, or from the cloud. The student can then see the results from the cloud through a web browser and can use the student application to interact with it and even reconfigure it remotely.

### 4.1. Instructor’s LabVIEW Front Panel Results

**Figure 15:**
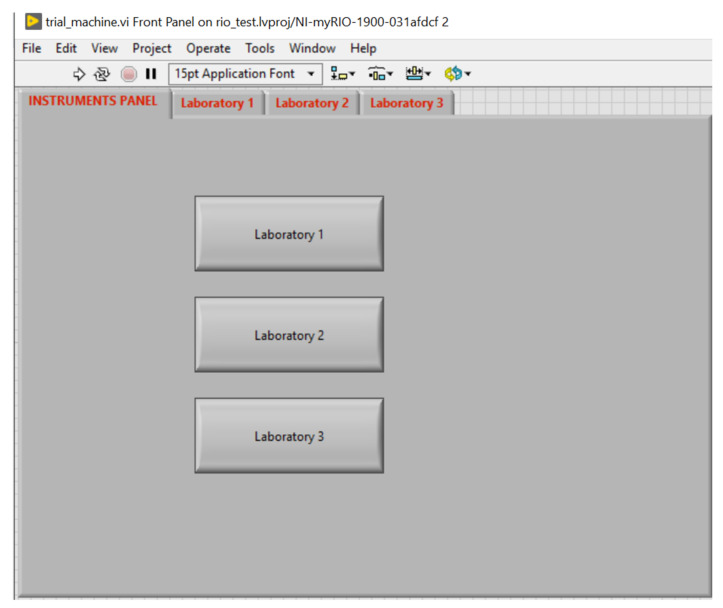
Front panel main tab.

**Figure 16:**
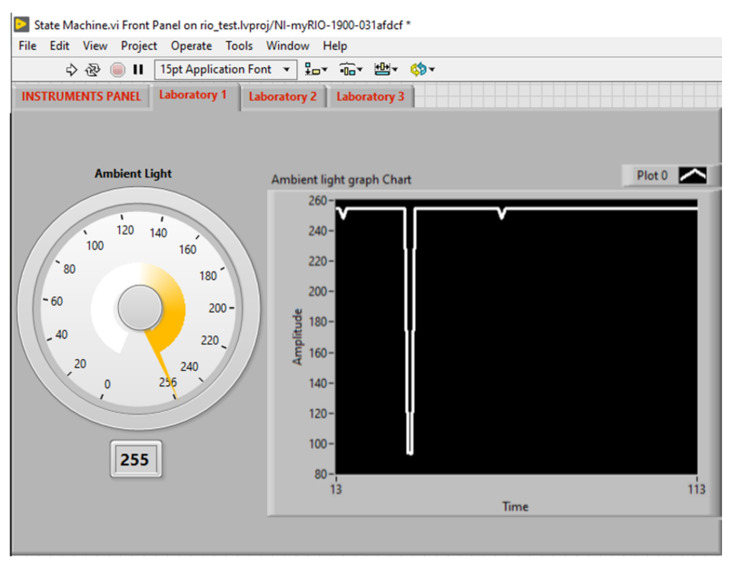
Laboratory 1 tab.

The front panel of the instructor’s LabVIEW application contains four tab elements, one main panel to choose from the laboratories, and three tabs that are used to acquire the values from the myRIO sensor node through Wi-Fi. Figure 14 illustrates the design of the front panel, which includes the following tabs:

- A tab to choose from the available laboratories (Figure 15).
- Laboratory 1 contains a knob displaying the value that comes from the ambient light sensor (Figure 16).
- Laboratory 2 includes a vertical slider showing the temperature measured by the temperature sensor.
- Laboratory 3 incorporate a horizontal slider displaying the range (in centimeters) measured by the infrared (IR) sensor.

When the user clicks a button on the first tab, the myRIO device will be reconfigured to acquire and display data accordingly. The values are also stored in global variables and sent to the cloud, where they can be read by both the instructor and the students.

**Figure 17:**
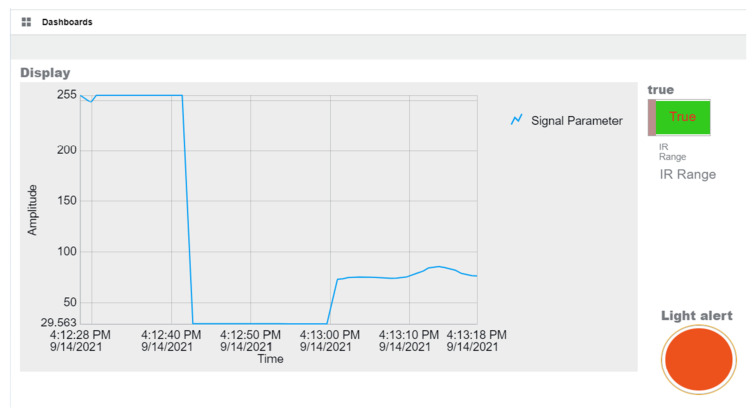
SystemLink Cloud display interface.

The values of the sensors from the sensor node are sent also to the cloud. Figure 17 shows the SystemLink Cloud web interface, which displays the values of the sensor on the graph. Depending on the state of the myRIO device, the axis of the graphic can automatically adjust to display either ambient light, temperature, or range value. The timeline for each sensor is shown in Table 4.

Furthermore, the configuration switch in the top right corner is used to reconfigure the learning platform and to select a particular sensor and appropriate LabVIEW application on the myRIO device.

This configurability allows a student to perform different experiments corresponding to the selected sensor and its application.

The light alert LED is used to show an alert when a specific threshold is exceeded for a particular sensor. The alert signal can be used to switch an actuator connected to the myRIO device over the Internet. This alert is generated from the remote student’s LabVIEW application.

### 4.2. Students’ Application

The students can connect to myRIO through the cloud by using a local LabVIEW Virtual Instrument. This is a simple program, which is remotely reading the value of the ambient light sensor and an alert led light will switch on or if the value read is under or above a set threshhold. The threshold can be adjusted with a knob according to requirements. The alert signal can be transmitted to switch a remote device.

By using this application, a student can gain the following skills:

➢ Configure the dashboard and setting up a tag in the cloud;
➢ Use LabVIEW to read the value from myRIO through the cloud;
➢ Experiment by modifying the application to accommodate different sensors and thresholds;
➢ Use the alert to switch on or off an external circuit;
➢ Learn to control devices remotely using LabVIEW;
➢ Customize the application for home or industrial control systems;
➢ Use LabVIEW to log data from myRIO through SystemLink Cloud.

**Figure 18:**
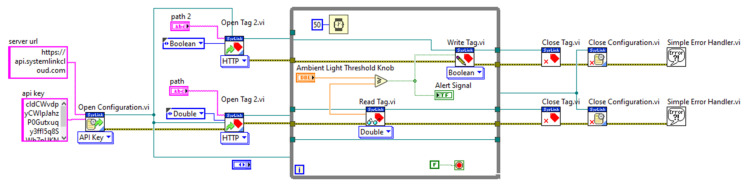
Block diagram for students’set-point application.

The code for the students’ application is illustrated in Figure 18. It performs read operations, to read the value of the ambient light sensor through the cloud and write operations, to write the alert to the cloud when the threshold is exceeded.

**Figure 19:**
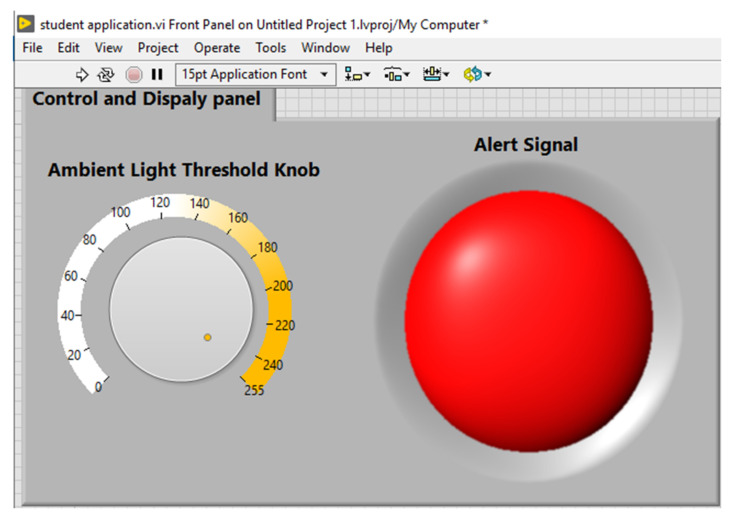
Front panel for students’ application.

The threshold can be set on the front panel by using the knob on the left side (Figure 19). The front panel also contains a round LED to display the alert (green means no alert, while the red color indicates an alert).

### 4.3. Discussion

Although the targets set out for this first phase of development have largely been achieved, there are of course some challenges and restrictions, but also certain weaknesses and functionalities that can be further developed or improved.

In electronic engineering, a well-developed laboratory involves validating concepts and theories [22]. This applies also to learning the proper use of sensors, which is a mandatory subject in electronics. We strongly believe our cloud-based laboratory will create a much better laboratory experience for the students, helping them in the educational process and providing reconfigurable laboratories. This environment provides an innovative learning experience, which will allow students to interact with the instructor.

Therefore, the main achievements and improvements to related remote laboratory platforms are the following:

- Design of a fully online laboratory platform, which can be easily accessed from anywhere and on any compatible device.
- The laboratory platform is reconfigurable either directly from the myRIO device, from the instructor’s PC, or from the cloud and can adapt automatically to run different sensor experiments.
- Student can access data from instructor’s device via the cloud and use them in their app.
- Student can remotely interact with instructor’s hardware platform and with the sensor node.
- Project has been done on NI ecosystem of myRIO, LabVIEW, and SystemLink Cloud, which ensures compatibility.
- By using the cloud, physical servers don’t need to be managed.
- The platform is highly scalable; hence it can accommodate high number of students at the same time.
- The secure access to the system is provided by SystemLink Cloud [23].

As already mentioned, the system has some limitations that can be improved in a further development phase. Table 5 describes the weaknesses found, also identifying a potential solution for them.

## 5. Conclusions

The current paper describes the design and development of a wireless configurable myRIO-based sensor node with cloud connectivity. Being forced into remote learning due to the ongoing COVID-19 pandemic, there is a pressing need for innovative learning solutions. In the first development phase of our investigation, three sensors have been used: the ambient light sensor, the proximity sensor, and the temperature sensor. The students can select which application to run on the learning platform and see the measurements either on the cloud via a web browser or on the local application. The innovation of our system is that it is software reconfigurable either from the instructor’s application, from the cloud, or directly from the device; it has a user-friendly interface; and it is easy to use. It can accommodate multiple applications and sensors and it is also accessible online, via a web browser, from anywhere and at any time. This environment creates a meaningful learning experience by allowing students to interact with the instructor. In this way, students learn software reconfigurability of hardware devices, and, in the future, they will be able to do further developments on their own. Through this blended learning, they will be able to discover and develop in electronics.

Furthermore, due to the ongoing COVID-19 pandemic, and taking into consideration that many of the university courses are now held online, the reliability and scalability of the system become critical. The platform developed meets these requirements as well, being highly available and scalable in the cloud.

In the context of remote education, we believe that our learning platform will be suitable for the COVID and the post-COVID era as well, considering that it provides a much better remote laboratory experience for students.

## Figures and Tables

**Figure 11:**
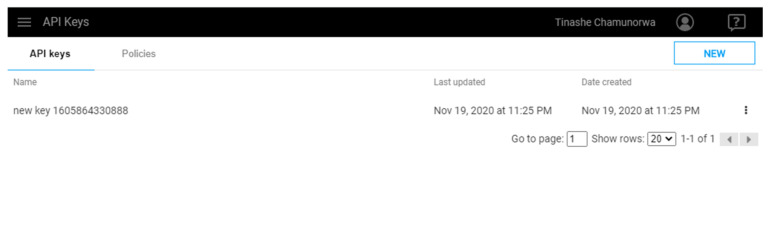
SystemLink Cloud API keys.

**Figure 12:**
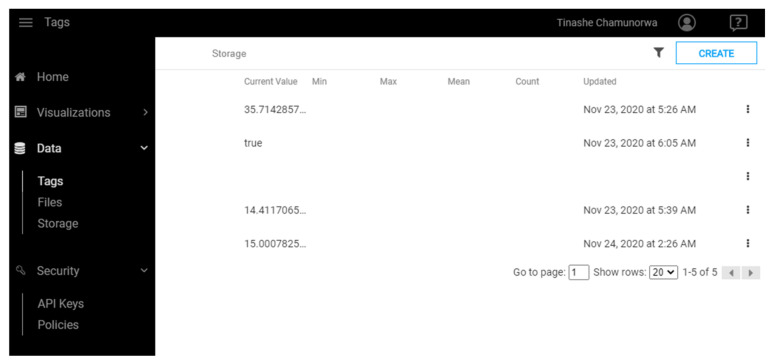
Tag configuration in SystemLink Cloud.

**Table 3:** Configurable SystemLink Cloud interface graphics.

Outputs	Decorations	Containers	Inputs
Gauge Widget	Image decoration	Tabs	Switch Widget
Chart/Scroll	Hyperlink text	iFrame widget	Horizontal/Vertical slider
XY/Graphs	Scalable Vector graphics	Template	Drop down list
LED (bool output) Widget	Dynamic Shapes	Template grid	Radio button group
Tank Widget	Free Text labels	Tabs	Check box

**Table 4:** Sensor values display timeline.

Parameter	Value	Time (PM)
Ambient light	~255	4:12:28–4:12:42
Temperature	~29.6	4:12:42–4:13:00
IR Range	~75	4:13:00–4:13:18

**Table 5:** Configurable SystemLink Cloud interface graphics.

Weakness	Solution	Status
Number of laboratories	accommodate more applications and more sensors	In development
Not expandable	use multiple myRIO devices in the same network	In development
Experiments	develop more applications in various field of electronics	In development
Connectivity	use of devices which connect directly to the Internet, without the need to use a computer	In analysis
Live view	add a live webcam for students	Considering

## Data Availability

The data presented in this study are available on request from the corresponding author.
